# Self-Reported Persistent Symptoms at 18 Months and Above Among COVID-19 Non-hospitalized Patients: A Prospective Cohort Study

**DOI:** 10.7759/cureus.43239

**Published:** 2023-08-09

**Authors:** Suman Kumar, Vipin Patidar, Shiv K Mudgal, Sanjay Kumar, Rajat Agarwal, Pratima Gupta, Rakhi Gaur, Saurabh Varshney

**Affiliations:** 1 Microbiology, All India Institute of Medical Sciences Deoghar, Deoghar, IND; 2 Nursing, All India Institute of Medical Sciences Deoghar, Deoghar, IND; 3 Anesthesiology and Critical Care, All India Institute of Medical Sciences Deoghar, Deoghar, IND; 4 Cardiothoracic Surgery, All India Institute of Medical Sciences Deoghar, Deoghar, IND; 5 Otolaryngology, All India Institute of Medical Sciences Deoghar, Deoghar, IND

**Keywords:** non-hospitalized, patients, persistent symptoms, infection, covid-19

## Abstract

Introduction: Since the beginning of the pandemic in early 2020, there have been numerous reports of symptoms that have lingered due to COVID-19. However, there is a lack of data concerning these persistent symptoms in non-hospitalized patients. This study sought to examine the prevalence of persistent symptoms at 18 months and beyond following the diagnosis of COVID-19 non-hospitalized patients.

Methods: A prospective cohort study comprised 212 non-hospitalized adult patients consecutively assessed from data available at tertiary care institutions through telephone interviews. During the interview, participants were routinely questioned about whether they were still experiencing any post-infection symptoms at the time of the study.

Results: Total 212 took part in the 18-month or longer follow-up survey. The most commonly reported symptoms during the acute phase were fever (n=149, 70.3%), weakness (n=118, 55.7%), and sore throat (n=100, 47.2%). At the 18-month and above follow-up, 167 patients (78.7%) reported at least one symptom continuing. The most common symptom at this time point was fatigue (n=109, 51.4%), followed by joint pain (n=57, 26.8%), and exertional dyspnea (24.5%). The possibility of symptoms returning after an 18-month follow-up and beyond was significantly lower in patients who had taken the COVID-19 vaccine (OR=0.29; 95% CI: 0.112-0.749; p=0.011) and those did not infect a second time (OR=0.232; 95% CI: 0.057-0.93; p=0.04).

Conclusion: The present study reveals that clinical complications persist even at 18 months and beyond during follow-up, with a prevalence similar to earlier follow-up periods, regardless of the severity of the initial COVID-19 infection.

## Introduction

Long COVID-19 is a complex condition that impacts various body systems and is characterized by persistent and severe symptoms following infection with the severe acute respiratory syndrome coronavirus 2 (SARS-CoV-2). Based on the assumption that 10% of people who are infected with COVID-19 will develop long COVID-19 and the fact that there have been over 651 million documented cases of COVID-19 globally, it is estimated that at least 65 million people worldwide are affected by long COVID-19 [1,2]. This number is likely an underestimate, as many cases of long COVID-19 may go undiagnosed. The occurrence of long COVID-19 varies across different groups, with estimates indicating 10%-30%, 50%-70%, and 10%-12% among non-hospitalized, hospitalized cases and among vaccinated individuals, respectively [3-5].

People of various ages and illness severity levels can develop long COVID-19 during the acute phase, with the highest incidence observed among individuals aged 36 to 50 years [5,6]. Since non-hospitalized patients with mild acute illnesses make up the bulk of COVID-19 cases overall, protracted COVID-19 are frequently identified in these patients [6].

Researchers have identified over 100 symptoms associated with COVID-19 after recovery, affecting various bodily systems [7]. Many reviews and meta-analyses have been conducted to assess the prevalence of these symptoms, but most of them featured follow-up intervals ranging from six to 12 months and incorporated data from patients who were hospitalized and those who were not [8-10]. Some experts have proposed that there may be a difference in the occurrence of post-COVID-19 symptoms in hospitalized versus outpatient populations [11]. A current meta-analysis reported a pooled prevalence of 34% (95% CI, 25%-46%) among patients who had not required hospitalization and 54% (95% CI, 44%-63%) among hospitalized patients supports this notion and also revealed that the prevalence of these symptoms differed according to the duration of a follow-up period utilized, albeit information for shorter follow-up periods was not included [12]. Another separate meta-analysis that focused on outpatients reported same rates of post-COVID-19 symptom prevalence among participants who were not hospitalized [13].

At present, there is limited data available on follow-up periods at 18 months and above after COVID-19, especially for non-hospitalized patients. As a result, the main aim of the present study was to investigate the prevalence of persistent COVID-19 symptoms and identify any potential risk factors associated with the persistence of post-COVID-19 symptoms in non-hospitalized COVID-19 patients during the follow-up period of 18 months or more after their acute infection.

## Materials and methods

Study design and participants

A cohort study was done from April 1 to May 15, 2023. Data available at a tertiary care institute were collected using a consecutive sampling of all adult non-hospitalized patients who tested positive for SARS-CoV-2 RNA through PCR testing of nasopharyngeal and throat swabs. The research received approval from the Institute Ethics Committees (2022-77-IND-02) and was conducted in adherence to the Helsinki Declaration. Prior to collecting any data, informed consent was taken from all participants.

Data collection methodology

Demographic information (age, sex, education, occupation, residence, marital status) and clinical details (COVID-19 symptoms at the beginning and related comorbidities) were compiled from medical records. Patients who agreed to take part in the studies were arranged for an expertly conducted telephone interview, scheduled 18 months or more after their acute COVID-19 infection (patients who were positive before September 2021). Participants were routinely questioned throughout the interview regarding the presence of symptoms that developed after the infection and if they remained at the time of the study.

The following criteria must be met in order for a symptom to be considered COVID-19 related: it must be directly related to the infection, not be brought on by any other underlying medical condition and appear within one month after the SARS-CoV-2 infection.

The study meticulously assessed the following post-COVID-19 symptoms: dyspnea (difficulty breathing), ageusia (loss of taste), fatigue, anosmia (loss of smell), hair loss, pain symptoms, diarrhea, palpitations, visual disorders, cough, brain fog (cognitive impairment) and loss of concentration. Additionally, participants were given the opportunity to report any other appropriate symptoms they felt.

Statistical analysis

The data were expressed as mean ±standard deviation or frequency (percentage), as applicable. The prevalence of symptoms was presented as a percentage of the total patients. To assess the likelihood of symptom persistence, odds ratios (OR) and their corresponding 95% confidence intervals (CI) were computed using a binary logistic regression model. Statistical analyses were performed using SPSS (Version 26; IBM Corp., Armonk, NY), and significance was determined at p < 0.05 (two-tailed).

## Results

Table 1 depicted that out of the 230 non-hospitalized patients who were invited to participate in the study, 212 of them (126 male (59.4%); a mean age of 38 (standard deviation of 13.2 years) took part in the 18-month or longer follow-up survey, resulting in a completion rate of 92.2%. The majority of the participants had completed their education up to graduation (42.5%), worked in healthcare professions (33.0%), were married (70.3%), and had been vaccinated (53.8%). Among the participants, 47 individuals (22.2%) reported having associated co-morbidities, with hypertension being the most common condition (n=26, 12.3%), followed by diabetes (n=12, 5.7%). The investigation period started 676 days after the diagnosis of COVID-19, with an interquartile range of 646.0 to 753.0 days.

**Table 1:** Demographic and clinical profile of non-hospitalized COVID-19 patients. N=Number of participants; SD: standard deviation; IQR: interquartile range; *Multiple response.

Characteristics (Demographic Profile)	N (%)
Age in years; Mean (SD)	38 (13.2)
Gender	
Male	126 (59.4)
Female	86 (40.6)
Education, N (%)	
Illiterate	14 (6.6)
Secondary	39 (18.4)
Graduation	90 (42.5)
Post-graduation/professionals	69 (32.5)
Occupation	
Housewife	27 (12.7)
Daily wages/Business/shopkeeper	41 (19.3)
Govt service	49 (23.1)
Healthcare professionals	70 (33)
Unemployed	25 (11.8)
Marital status	
Single	58 (27.4)
Married	149 (70.3)
Divorced/separated/Widow	5 (2.4)
Family income	
Less than 10,000/-	24 (11.3)
10,000/- to 20,000/-	39 (18.4)
20,000/- to 30,000/-	39 (18.4)
More than 30000/-	110 (51.9)
Residence	
Urban	127 (59.9)
semi-urban	31 (14.6)
Rural	54 (25.5)
Days from COVID-19 report were positive to survey, median (IQR)	676 (646.0-753.0)
Vaccination status before COVID-19 infection	
Yes	114 (53.8)
No	98 (46.2)
Presence of comorbidity	
No	165 (77.8)
Yes	47 (22.2)
Comorbidity	
Diabetes	12 (5.7)
Hypertension	26 (12.3)
Respiratory illness (COPD, Asthma)	3 (1.4)
Others (Thyroid disease, kidney disease)	6 (2.8)
Second infection with COVID-19	
Yes	44 (20.8)
No	168 (79.2)
Symptoms of COVID-19 during hospital visit*	
Fever	149 (70.3)
Loss of smell	71 (33.5)
Backpain	51 (24.1)
Weakness	118 (55.7)
Frequent coughing	68 (32.1)
Sore throat	100 (47.2)
Joint pain	43 (20.3)
Anorexia	25 (11.8)
Loss of taste	44 (20.8)
Nausea	26 (12.3)
Shortness of breath	38 (17.9)
Headache	18 (8.5)

Table 1 also present the prevalence of symptoms during the acute phase of COVID-19. The most commonly reported symptoms during the acute phase were fever (n=149, 70.3%), weakness (n=118, 55.7%), sore throat (n=100, 47.2%), loss of smell (n=71, 33.5%%), and frequent cough (n=68, 32.1%).

**Figure 1:**
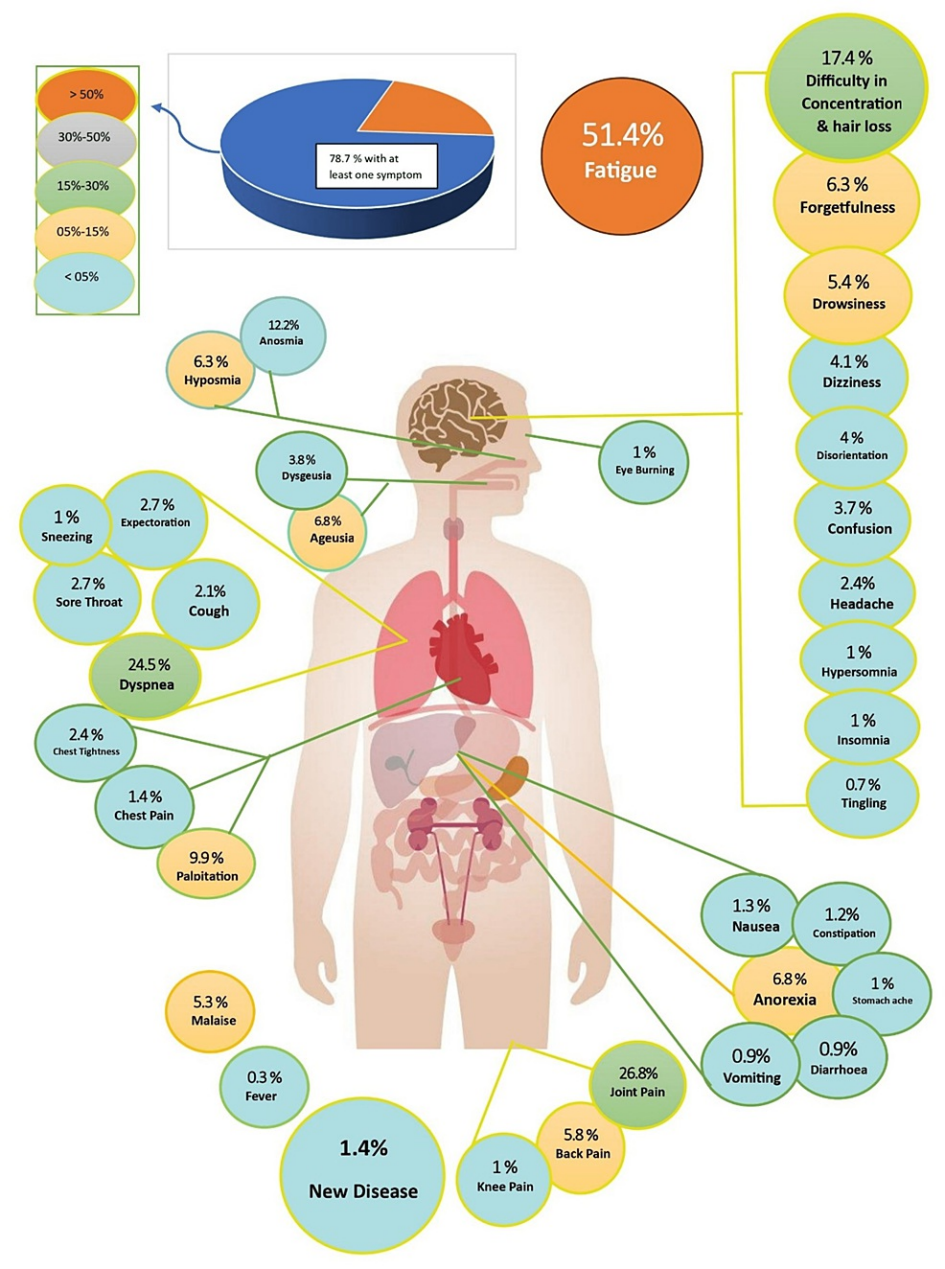
Persistent symptoms at 18 months and above after COVID-19 infection Credit: Authors

At the 18-month and above follow-up, 167 patients (78.7%) noted that at least one symptom had stayed. The most frequently persistent symptom at this time point was fatigue (n=109, 51.4%), followed by joint pain (n=57, 26.8%), with 52 patients experiencing exertional dyspnea (24.5%) and 37 patients experiencing difficulty in concentration and hair loss (17.4%). Other persistent symptoms included anosmia (n=26, 12.2%) and palpitation (n=21, 9.9%) (Figure 1).

Table 2 presented that the possibility of symptom constancy at the 18-month and above after acute infection was slightly greater in women (OR=3.14; 95% CI: 1.02-9.68; p=0.046), in individuals residing in semi-urban (OR=1.57; 95% CI: 2.01-7.26; p=0.01) and rural (OR=2.30; 95% CI: 1.10-9.83; p=0.03) and those who had any comorbidity (OR=5.008; 95% CI: 1.47-16.97; p=0.01). The symptoms persistence at 18 months and above follow-up was significantly lower in patients who had taken COVID-19 vaccine (OR=0.29; 95% CI: 0.112-0.749; p=0.011) and those not infected second time (OR=0.232; 95% CI: 0.057-0.93; p=0.04).

**Table 2:** Odd ratio for symptoms persistence at 18-month and above follow-up according to demographic and clinical profile of non-hospitalized COVID-19 patients (N=212). OR: Odd ratio; CI: Class interval; N: Number of participants

	Yes		No		OR (95% CI)	P-value
	N	(%)	N	(%)		
Age in years						
<30	67	77.9	19	22.1	Reference	
31-45	54	79.4	14	20.6	0.814 (0.233-2.84)	0.748
>46	46	79.3	12	20.7	0.641 (0.12-3.38	0.6
Gender						
Male	95	75.4	31	24.6	Reference	
Female	72	83.7	14	16.3	3.14 (1.02-9.68)	0.046
Education						
Illiterate	11	78.6	3	21.4	Reference	
Secondary	32	82.1	7	17.9	1.59(0.20-12.38)	0.655
Graduation	68	75.6	22	24.4	0.64 (0.69-5.92)	0.695
Post-graduation/professionals	56	81.2	13	18.8	0.98 (0.83-11.70)	0.99
Occupation						
Housewife	21	77.8	6	22.2	Reference	
Daily wages/Business/shopkeeper	32	78.0	9	22	2.41 (0.36-15.84)	0.358
Govt service	41	83.7	8	16.3	3.26 (0.33-32.00)	0.31
Healthcare professionals	54	77.1	16	22.9	1.69 (0.19-14.82)	0.632
Unemployed	19	76.0	6	24	2.01 (0.24-16.53)	0.514
Marital status						
Single	44	75.9	14	24.1	Reference	
Married	119	79.9	30	20.1	1.06 (0.28-4.03)	0.921
Divorced/separated/Widow	4	80.0	1	20	0.31 (0.01-5.16)	0.417
Family Income						
Less than 10,000/-	18	75.0	6	25	Reference	
10,000/- to 20,000/-	29	74.4	10	25.6	0.60 (0.10-3.34)	0.562
20,000/- to 30,000/-	31	79.5	8	20.5	1.66 (0.30-9.11)	0.56
More than 30,000/-	89	80.9	21	19.1	1.66 (0.26-10.53)	0.586
Residence						
Urban	93	73.2	34	26.8	Reference	
semi-urban	30	96.8	1	3.2	1.57 (2.01-7.26)	0.01
Rural	44	81.5	10	18.5	2.30 (1.10-9.83)	0.03
Vaccination status before COVID-19 infection						
Yes	93	81.6	21	18.4	Reference	
No	74	75.5	24	24.5	0.29 (0.112-0.749)	0.011
Presence of comorbidity						
No	123	74.5	42	25.5	Reference	
Yes	44	93.6	3	6.4	5.008 (1.47-16.97)	0.01
Second infection with COVID-19						
Yes	41	93.2	3	6.8	Reference	
No	126	75.0	42	25	0.232 (0.057-0.93)	0.04

## Discussion

This is the first cohort study that we are aware of, investigating the existence of persistent COVID-19 symptoms in non-hospitalized patients 18 months or more after their infection. Previous information on non-hospitalized patients has been based on follow-up periods not exceeding six and 12 months [14-20], making it challenging to directly compare our results with those earlier findings. However, earlier studies have suggested decreased prevalence rates of long COVID-19 symptoms among patients who were not hospitalized in comparison to hospitalized patients [14-20].

In our research, we found that 78.7% of patients continue to experience persistent COVID-19-related symptoms, which was higher compared to a previous study reporting 52.7% in mild COVID-19 patients after a 12-month follow-up [14]. This disparity in frequency could be attributed to the broader range of persistent symptoms we included in our study and the variation in follow-up durations. Given that our investigation specifically focused on non-hospitalized patients, the prevalence of long-lasting COVID-19 symptoms, as indicated by our results, is a matter of significant concern.

In our study, various persistent symptoms were observed, with the most prevalent ones being fatigue, joint pain, exertional dyspnea, difficulty in concentration, hair loss, and anosmia. These symptoms were consistent with those reported in previous studies [21-23] at 12-month follow-up. These results provide evidence that fatigue is likely the most widespread and persistent symptom after COVID-19. A recent study suggested that post-COVID-19 fatigue is comparable to chronic fatigue syndrome or myalgic encephalomyelitis. Chronic fatigue syndrome, myalgic encephalomyelitis, and extended COVID-19 have all been connected to similar endothelial impairment [24].

The possible mechanism behind the emergence of neuropsychological symptoms was explained, linking it to inflammation-induced disruptions in the blood-brain barrier. This disruption permits an increased flow of cytokines into the brain. The resulting neuroinflammation, triggered by microglial activation and oxidative stress, may play a role in short-term delirium and long-term cognitive and functional decline [25].

Several investigations have reported the prevalence of anosmia and ageusia after 12 months of COVID-19 recovery. In one study [15], it was found that 27.3% of patients, with a median age of 72 years, experienced these symptoms. However, in another study that included patients with a median age of 37 years [14], 94.1% of the patients had objectively recovered from anosmia after the same 12-month period. In our own study, participants had a mean age of 38 years and found that only 12.2% of the total respondents still presented with anosmia. These results further support the notion that complete olfactory recovery is more likely in younger individuals [23].

It is crucial to identify risk factors that can help predict the development of long COVID-19, the duration of symptoms, and whether COVID-19 may lead to the emergence of other chronic conditions. However, information on these aspects in non-hospitalized patients is limited, as far as we know. Previous studies [11,14] have identified some phenotypes in non-hospitalized COVID-19 patients, but their conclusions were based on follow-up periods ranging from six to 12 months post-infection. Some research suggests that being female and having associated comorbidities might be potential risk factors for long COVID-19.

In our study, we found that being female, residing in rural or semi-urban areas, and having comorbidities during the acute phase of the infection were risk factors for persistent COVID-19 symptoms among non-hospitalized patients. These findings align with previous research [11,14], which reported that female sex and comorbidities were associated with long COVID-19 symptoms. Our study also revealed that women might be more vulnerable than males to the effects of chronic symptoms. It is good that artificial intelligence can be utilized for assessing and managing long COVID-19 symptoms [26].

This study represents the COVID-19 persistent symptoms among non-hospitalized patients after an 18-month and above period since the infection. However, it is essential to approach the current data cautiously and take into account its limitations. To begin with, the lack of a control group could affect how we interpret the results. The study's single-center methodology and small sample size could also limit its applicability to a broader population. The absence of hospitalized patients from the study is the third limitation, which underscores the need for more research comparing the long-term outcomes of inpatients and outpatients while also making the sample more comparable. Lastly, it is worth mentioning that the symptoms were reported by the patients themselves and gathered through telephonic interviews.

Notwithstanding these constraints, our findings underscore the urgency of improving the readiness and proficiency of healthcare practitioners in recognizing and handling the persistent symptoms associated with the long COVID-19. Additionally, this study emphasizes the significance of future research into persistent symptoms following COVID-19 infection in vaccinated individuals. This is because persistent symptoms can endure for up to 18 months and above after recovery from COVID-19 infection, even in individuals who are non-hospitalized.

## Conclusions

In summary, based on a group of confirmed COVID-19 cases involving patients who did not require hospitalization, our study reveals that clinical complications persist even at 18 months and beyond during follow-up, with a prevalence similar to earlier follow-up periods, regardless of the severity of the initial COVID-19 infection. It is crucial to identify patients at risk to facilitate management, which will ultimately lead to better results and reduced healthcare expenses. The data obtained from our research will be valuable in advocating for health policy measures, including long-term surveillance programs and specialized facilities designed to address the needs of individuals affected by long COVID-19 syndrome.
